# Genome‐wide SNP analysis unveils genetic structure and phylogeographic history of snow sheep (*Ovis nivicola*) populations inhabiting the Verkhoyansk Mountains and Momsky Ridge (northeastern Siberia)

**DOI:** 10.1002/ece3.4350

**Published:** 2018-07-16

**Authors:** Arsen V. Dotsev, Tatiana E. Deniskova, Innokentiy M. Okhlopkov, Gabor Mészáros, Johann Sölkner, Henry Reyer, Klaus Wimmers, Gottfried Brem, Natalia A. Zinovieva

**Affiliations:** ^1^ L.K. Ernst Federal Science Center for Animal Husbandry Moscow Region Podolsk Russian Federation; ^2^ Institute for Biological Problems of Cryolithozone Yakutsk Russian Federation; ^3^ Division of Livestock Sciences University of Natural Resources and Life Sciences Vienna Austria; ^4^ Institute of Genome Biology Leibniz Institute for Farm Animal Biology (FBN) Dummerstorf Germany; ^5^ Institute of Animal Breeding and Genetics University of Veterinary Medicine (VMU) Vienna Austria

**Keywords:** genetic diversity, *Ovis nivicola*, population structure, single‐nucleotide polymorphisms, snow sheep

## Abstract

Insights into the genetic characteristics of a species provide important information for wildlife conservation programs. Here, we used the OvineSNP50 BeadChip developed for domestic sheep to examine population structure and evaluate genetic diversity of snow sheep (*Ovis nivicola*) inhabiting Verkhoyansk Range and Momsky Ridge. A total of 1,121 polymorphic SNPs were used to test 80 specimens representing five populations, including four populations of the Verkhoyansk Mountain chain: Kharaulakh Ridge–Tiksi Bay (TIK,* n *=* *22), Orulgan Ridge (ORU,* n *=* *22), the central part of Verkhoyansk Range (VER,* n *=* *15), Suntar‐Khayata Ridge (SKH,* n *=* *13), and Momsky Ridge (MOM,* n *=* *8). We showed that the studied populations were genetically structured according to a geographic pattern. Pairwise *F*_ST_ values ranged from 0.044 to 0.205. Admixture analysis identified *K *=* *2 as the most likely number of ancestral populations. A Neighbor‐Net tree showed that TIK was an isolated group related to the main network through ORU. TreeMix analysis revealed that TIK and MOM originated from two different ancestral populations and detected gene flow from MOM to ORU. This was supported by the f3 statistic, which showed that ORU is an admixed population with TIK and MOM/SKH heritage. Genetic diversity in the studied groups was increasing southward. Minimum values of observed (Ho) and expected (He) heterozygosity and allelic richness (Ar) were observed in the most northern population—TIK, and maximum values were observed in the most southern population—SKH. Thus, our results revealed clear genetic structure in the studied populations of snow sheep and showed that TIK has a different origin from MOM, SKH, and VER even though they are conventionally considered a single subspecies known as Yakut snow sheep (*Ovis nivicola lydekkeri*). Most likely, TIK was an isolated group during the Late Pleistocene glaciations of Verkhoyansk Range.

## 1 INTRODUCTION

**Figure 1:**
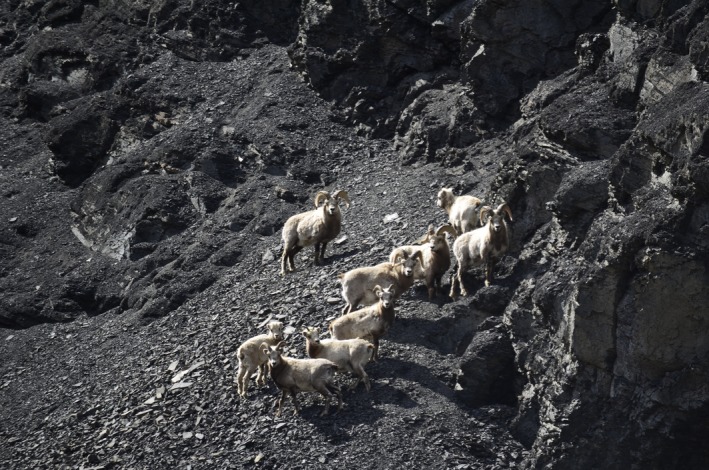
A group of snow sheep in their natural habitat

Snow sheep or Siberian bighorn sheep (*Ovis nivicola* Eschscholtz, 1829) (Figure 1) inhabits the most northern territories among all Asian ovine species, from Putorana Plateau in the east to Chukotka Peninsula in the west and from the Okhotsk Sea shore in the south to the Arctic Ocean shore in the north. As the only large herbivorous mammal in the mountainous regions of northeast Russia, snow sheep plays an important role in the ecosystems of the region, the main features of which are low resilience, vulnerability, and low self‐restoration ability of the environment. The snow sheep habitat is located in several natural zones with different types of climate and environmental conditions. Despite the fact that the snow sheep has a high tolerance to different climatic conditions, it has not been able to survive the environmental changes caused by anthropogenic factors. In almost all the mountain systems, the snow sheep has been displaced by human activities, including mining of minerals, road laying, development of industrial areas, and intensive management of domestic reindeer (Zheleznov‐Chukotsky, 1994). The fact that many subspecies are found in high mountain regions is the result of long‐term anthropogenic pressure. This is confirmed by the evidence that several populations, which were abundant in lowlands, coastal areas, and middle‐altitude mountains a few decades ago, now do not exist. Consequently, the snow sheep range is currently discontinuous and consists of several fragmented territorial units in various mountain systems (Zheleznov‐Chukotsky, 2007). In 2016, the total number of snow sheep was estimated to be 78 thousand individuals (Ministry of Natural Resources and Environment of the Russian Federation, http://www.mnr.gov.ru/opendata/7710256289-hunting-resources). The largest units are located in Yakutia, Kamchatka, and Okhotsk Seaboard. However, even the sheep in those areas are decreasing, especially at the periphery. A few years ago, the Putorana and Chukchi subspecies received a special conservation status—they are now listed in the Red Book of the Russian Federation (Zheleznov‐Chukotsky, 2007). Therefore, in order to characterize the importance of individual populations for the survival and adaptability of the species and to derive conservation measures, it is necessary to study its genetic diversity. Higher genetic diversity provides species with a greater ability to adapt to changes in the environment (Campbell et al., 2009; Mergeay & Santamaria, 2012).

The most promising method for the investigation of genetic diversity and population structure is the analysis of single‐nucleotide polymorphisms (SNPs). SNP genotyping is based on a substitution in a single nucleotide at a specific position in the genome (Vignal, Milan, SanCristobal, & Eggen, 2002). This technique provides reproducible results in different laboratories without any standardization of protocols or adjustment of raw data (Morin, Luikart, Wayne, & the SNP workshop group, 2004; Vignal et al., 2002). Recent advances in the development of high‐throughput genotyping platforms have turned SNPs into a powerful tool for population and genetic studies of domestic animals (Coates et al., 2009; Kijas et al., 2009).

The creation of SNP arrays for wild animals would be an important step for better understanding their phylogenetic relationships and genetics in general (Miller, Kijas, Heaton, McEwan, Coltman, 2012; Seeb et al., 2011) and could be used in wildlife forensics (Ogden, 2011). However, the absence of reference genomes and expensiveness and unprofitability are the limiting factors for such developments.

As a solution to this problem, it was suggested that the cross‐species application of commercially available SNP chips designed for domestic animals would be beneficial in studies on their respective wild relatives, despite the fact that the number of polymorphic loci decreases (Kohn, Murphy, Ostrander, & Wayne, 2006; Miller, Poissant, Kijas, Coltman, & the International Sheep Genomics Consortium, 2011).

Thus, Tokarska et al. (2009) evaluated the population structure of European bison *(Bos bonasus)* using the BovineSNP50 BeadChip created for cattle *(Bos taurus)*. With the same chip, Haynes and Latch (2012) differentiated indigenous species of deer in North America, including mule deer and black‐tailed deer (*Odocoileus hemionus*) and white‐tailed deer (*Odocoileus virginianus*). Kharzinova et al. (2015) have shown the applicability of the BovineSNP50 BeadChip for whole‐genome study of reindeer *(Rangifer tarandus)*. Miller et al. (2011) performed SNP genotyping of two wild *Ovis* species, bighorn (*O. canadensis*) and thin‐horn sheep (*O. dalli*), using the OvineSNP50 BeadChip.

The aim of our study was to investigate the genetic diversity and population structure of snow sheep (*O. nivicola*) and to obtain information that could be used in the development of conservation strategies and as a first step in understanding the taxonomy and evolution of snow sheep based on genotypes.

## 2 MATERIALS AND METHODS

### 2.1 Ethics statement

Muscle tissue samples of snow sheep were collected under permits issued by the Department of Hunting of the Republic Sakha (Yakutia) during scientific expeditions. Some samples were taken from indigenous peoples’ representatives, who are licensed to hunt snow sheep for personal consumption according to the Federal Law of the Russian Federation.

### 2.2 Sample collection

**Figure 2:**
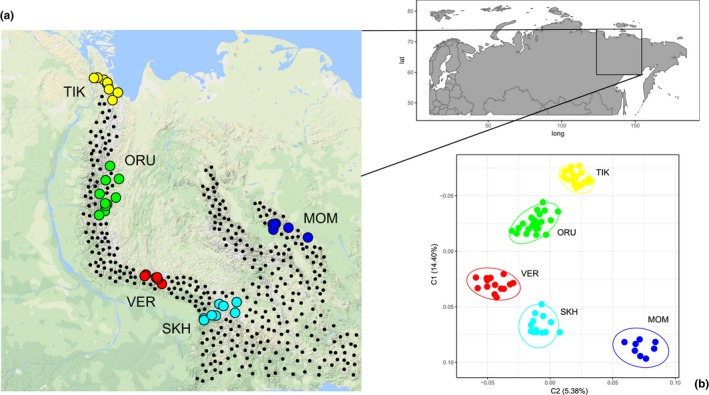
Map of the sampling sites of the snow sheep individuals included in the study (a) and their clustering in a multidimensional scaling (MDS) analysis based on an identity‐by‐state (IBS) matrix (b). The area of the distribution of snow sheep in the Verkhoyansk Mountains and Momsky Ridge is marked with dots

The sampling sites of individual snow sheep are presented in Figure 2a. The specimens selected for analysis (*n *=* *80) were collected in different parts of the Republic Sakha (Yakutia), Russia, covering the area between 63 and 71 degrees north latitude and between 128 and 146 east longitude during 2011–2016. They were divided into five groups according to their geographic locations. Four populations represented the Verkhoyansk Mountain chain including Kharaulakh Ridge at Tiksi Bay (TIK, *n *=* *22), Orulgan Ridge (ORU, *n *=* *22), the central part of Verkhoyansk Range (VER, *n *=* *15), and Suntar‐Khayata Ridge (SKH, *n *=* *13). The fifth population included sheep inhabiting Momsky Ridge (MOM, *n *=* *8).

### 2.3 DNA extraction and SNP genotyping

Genomic DNA was extracted from muscle tissue samples using a Nexttec column (Nexttec Biotechnology GmbH, Germany), following the manufacturer's instructions. The DNA concentration was estimated by measuring the absorbance at 260 nm, and the DNA quality was checked by separation on agarose gels. SNP genotyping was performed using the OvineSNP50 BeadChip, which was developed by Illumina (Illumina, San Diego, CA, USA) in collaboration with the International Sheep Genomics Consortium (ISGC) (Kijas et al., 2009, 2012; OvineSNP50 Genotyping BeadChip. https://www.illumina.com/documents/products/datasheets/datasheet_ovinesnp50.pdf). The BeadChip contains more than 54,000 SNPs that cover the entire ovine genome and therefore enables a broad range of applications, including genomewide selection, identification of quantitative trait loci (QTL), evaluation of genetic merit, crossbreed mapping, linkage disequilibrium studies, comparative genetic studies, and breed characterization for evaluating biodiversity.

### 2.4 Quality control and statistical analysis

#### 2.4.1 Quality control

To select SNPs to create a set of markers suitable for snow sheep from the chip designed for domestic sheep (Illumina OvineSNP50 BeadChip), quality control was performed. During an initial quality check, GenCall (GC) and GenTrain (GT) cutoff scores of 0.5 were applied to determine the valid genotypes for each SNP. The GC score, or no‐call threshold, is a quality metric representing the accuracy of genotyping, and the GT score is a statistical measure from the Illumina clustering algorithm (Infinium genotyping data analysis. A guide for analyzing Infinium genotyping data using the GenomeStudio Genotyping Module. https://www.illumina.com/Documents/products/technotes/technote_infinium_genotyping_data_analysis.pdf; Illumina GenCall data analysis software. GenCall software algorithms for clustering, calling, and scoring genotypes. https://www.illumina.com/Documents/products/technotes/technote_gencall_data_analysis_software.pdf).

Quality filtering of genetic markers was performed in PLINK v1.9 (Purcell et al., 2007). SNPs located on sex chromosomes and SNPs with unknown map positions were deleted. SNPs that were genotyped in less than 90% of individuals (−geno 0.1) with a minor allele frequency (MAF) < 1% (–maf 0.01) and in linkage disequilibrium (–indep‐pairwise 50 5 0.5) were pruned. For estimation of SNPs in LD, the LD between each pair of SNPs in a window of 50 SNPs was calculated, and one of a pair of SNPs was removed if the LD was >0.5. Then, the window was shifted 5 SNPs forward, and the procedure was repeated. Snow sheep individuals were initially quality‐controlled in PLINK v1.9; the ones that had less than 90% of SNPs genotyped (–mind 0.1) were removed. Afterward, animals were tested for their relationships. Relatedness between the individuals was identified in ML‐Relate software (Kalinowski, Wagner, & Taper, 2006). One of the animals (chosen randomly) from a full‐sib pair was removed.

### 2.5 Statistical analyses

The observed (*H*_o_) and unbiased expected (*H*_e_) heterozygosity (Nei, 1978), inbreeding coefficient (*F*_IS_), and rarified allelic richness (Ar) were calculated in the R package “diveRsity” (Keenan, McGinnity, Cross, Crozier, & Prodöhl, 2013) for each studied population separately and for all the groups as a whole. Pairwise fixation indices (*F*_ST_) (Weir & Cockerham, 1984) and their respective *p*‐values were calculated in the R package “StAMPP” (Pembleton, Cogan, & Forster, 2013).

Multidimensional scaling (MDS) based on a pairwise identity‐by‐state (IBS) distance matrix was performed with PLINK 1.9 (–genome, –cluster, –mds‐plot 4) and visualized in the R package “ggplot2” (Wickham, 2009).

Population structure was evaluated using Admixture 1.3 software (Alexander, Novembre, & Lange, 2009). The program was run from *K *=* *1 to *K *=* *7 to determine the lowest cross‐validation (CV) error, which shows the optimal number of clusters. The individuals within populations were ordered according to the sampling points based on the following: TIK and ORU by latitude from north to south, VER from northwest to southeast, and SKH and MOM by longitude from west to east. The output files were visualized using the R package “pophelper” (Francis, 2017). The assignment percentage of the studied populations to the clusters was given as average ancestry coefficients (*q*‐values).

An individual tree based on the pairwise identity‐by‐state (IBS) distance matrix (–distance 1‐ibs) and a population tree based on the pairwise *F*_ST_ values were constructed using the Neighbor‐Net algorithm implemented in SplitsTree 4.14.5 (Huson & Bryant, 2006).

To estimate and visualize the distribution of heterozygosity at the individual level, we also calculated standardized multilocus heterozygosity (sMLH) in the R package “inbreedR” (Stoffel et al., 2016). sMLH (Coltman, Pilkington, Smith, & Pemberton, 1999) is defined as the total number of heterozygous loci in an individual divided by the sum of the average observed heterozygosities in the population over the subset of loci successfully typed in the focal individual.

A Mantel test (Mantel, 1967 **)** was performed in the software GenAlEx 6.5 (Peakall & Smouse, 2012). The geographic coordinates for the groups were calculated as the average latitude/longitude of all relevant sampling points using the function geomean in the R package geosphere (Hijmans, 2017). The function distGeo of this R package was used for the calculation of geographic distances (km) between the groups. Due to the U‐shaped habitat area of the studied populations, the geographic distribution was calculated as the sum of distances between neighboring groups. Pairwise *F*_ST_ values were used as genetic distances for the test. Data used for the Mantel test are presented in Supporting Information Table S1.

TreeMix 1.13 (Pickrell & Pritchard, 2012) was used to construct maximum‐likelihood trees that best described the relationship among the populations and to infer migration events between them. This software was also used for the calculations of f3 statistics (Reich, Thangaraj, Patterson, Price, & Singh, 2009). Samples of Tien‐Shan argali (*Ovis ammon karelini*) from Kyrgyzstan were used as an out‐group (OUT, *n *=* *6) for the TreeMix analysis. The selection of the out‐group was limited to the species that could be genotyped using the same SNP BeadChip, that is, belonging to the genus *Ovis*. We selected *O. ammon* because its habitat was the closest to *O. nivicola* of all Eurasian wild species and because they could have a common ancestor. The divergence time between the two species was estimated to be 2.4–3.1 MYA (Bunch, Wu, Zhang, & Wang, 2006; Rezaei et al., 2010). To integrate the two datasets, we selected only the loci (1121 SNPs) that were used for the snow sheep analyses. Examination of these markers in the *O. ammon* dataset revealed that two SNPs were not called, and these SNPs were therefore excluded from the merged dataset.

The geographic map (with longitude and latitude coordinates for each sampling site) was plotted using the R packages maps and mapdata (Becker, Wilks, Brownrigg, Minka, & Deckmyn, 2017). The map with overlaying admixture pie charts was constructed using the R packages LEA (Frichot & François, 2015) and mapplots (Gerritsen, 2014).

## 3 RESULTS

After the relatedness test in which one individual from a full‐sib pair was removed, a total of 80 individuals with at least 90% genotyped SNPs were included in this study. From the initial set of 54,241 SNPs available in the Illumina OvineSNP50 BeadChip, we selected for subsequent analyses 1,121 polymorphic loci (~2%), which passed all the quality control procedures. The selected SNPs were distributed over all chromosomes, and their number ranged from 15 on chromosome 22–124 on chromosome 1 (Supporting Information Figure S1a). The SNP ratio, which was calculated as the number of selected SNPs for snow sheep divided by the number of SNPs on the respective chromosome, ranged from 0.014 to 0.036 (Supporting Information Figure S2b).

### 3.1 Clustering of the individual samples

To examine the relationship among individuals, we performed a multidimensional scaling (MDS) analysis and constructed a Neighbor‐Net tree based on an identity‐by‐state (IBS) matrix. The MDS analysis showed variances of 14.40% and 5.38% in the first and the second components (C1 and C2), respectively (Figure 2b). The first component divided the studied populations into two groups: northeastern (C1 < 0, TIK and ORU) and southeastern (C1 > 0, VER, SKH, and MOM). All snow sheep sampled from five geographic regions were correctly assigned to their origin. Similar results were shown in the Neighbor‐Net tree; all the individual branches were grouped with the clades representing their respective populations (Supporting Information Figure S2). The most distant populations, TIK and MOM, were positioned on the opposite sides of the tree. ORU formed two clusters, which separated TIK from the other clades.

### 3.2 Population structure

**Figure 3:**
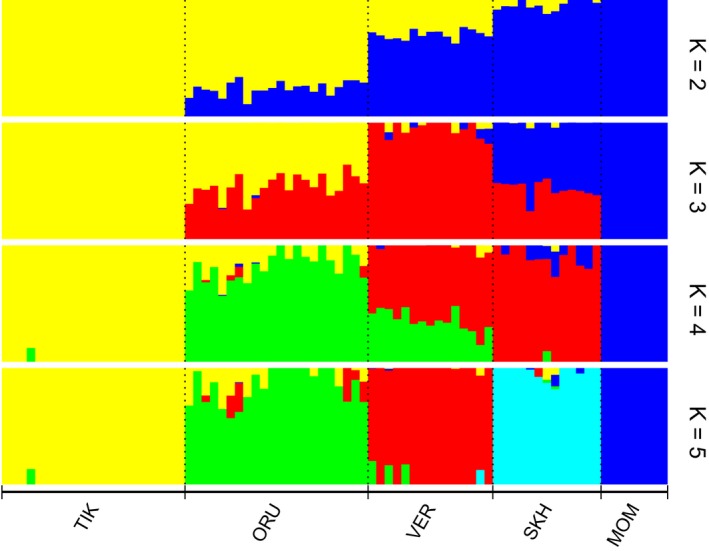
Population structure of snow sheep by model‐based clustering using Admixture 1.3 software. The results from *K *=* *2 to *K *=* *5 are shown

To estimate the proportion of common ancestry among the populations of snow sheep, a clustering analysis was performed with Admixture 1.3 software (Figure 3) for values of K from 1 to 7. The lowest cross‐validation (CV) error, showing the assumed number of populations, was observed for *K *=* *2 (Supporting Information Figure S3). At *K *=* *2, clear differentiation of TIK from MOM and SKH was observed. For VER and ORU, a high level of admixture with different proportions was shown. At *K *=* *5, all the populations were clearly separated from each other.

### 3.3 Genetic differentiation

To estimate the level of genetic differentiation, pairwise *F*_ST_ values were calculated (Supporting Information Table S1). Higher *F*_ST_ values were observed between the most geographically distant pairs: TIK and MOM (*F*_ST_ = 0.205) and ORU and MOM (0.142). Between adjacent populations, the values were lowest for TIK and ORU (0.052), ORU and VER (0.053), and VER and SKH (0.044). All the *F*_ST_ values were highly significant (*p* < 0.001).

**Figure 4:**
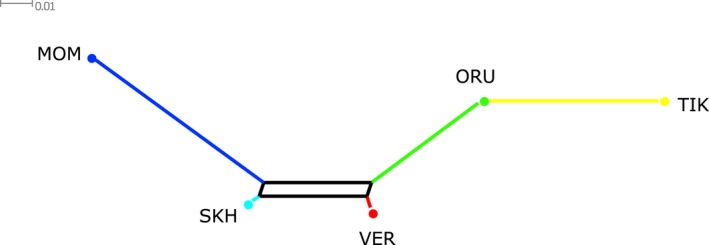
Neighbor‐Net tree based on pairwise *F*_ST_ distances

The Neighbor‐Net analysis based on pairwise *F*_ST_ distances depicted a network representing the phylogenetic relationships between the studied populations (Figure 4). It was shown that while MOM, SKH, VER, and ORU are all related to each other in the tree, TIK was an isolated group, related only to ORU.

**Figure 5:**
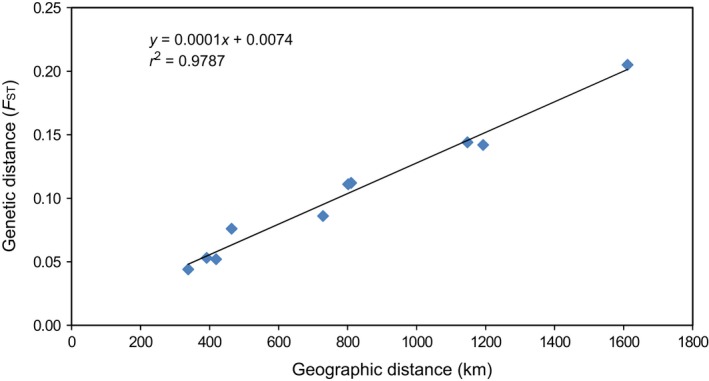
Mantel test showing the relationship between genetic (pairwise *F*_ST_) and geographic (km) distances in the populations of snow sheep

To evaluate the isolation by distance (IBD), a Mantel test was conducted (Figure 5). The analysis, based on 9999 permutations, demonstrated a significant correlation (*r* ^2^ = 0.979) between the genetic (*F*_ST_) and geographic (km) distances (*p* = 0.009).

### 3.4 Phylogeographic analysis

**Figure 6:**
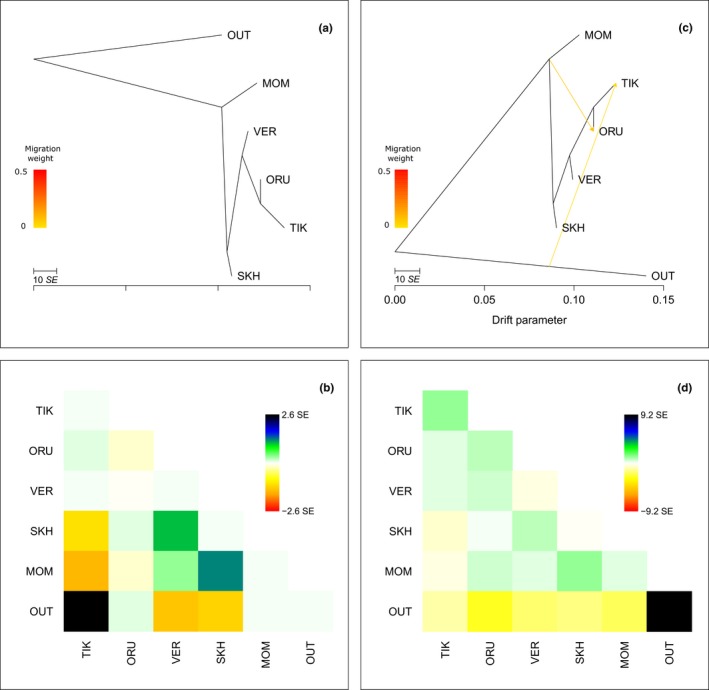
Most frequently found maximum‐likelihood trees inferred from five snow sheep (*Ovis nivicola*) populations and argali (*Ovis ammon*) set as an out‐group (OUT) with no migration events (a) with its respective residual fit (b) and with two migration events (c) with its respective residual fit (d)

To examine historical relationships among the studied populations, a TreeMix analysis was performed. For this reason, we merged our dataset of 1121 snow sheep polymorphic SNPs with the genotyping profiles of argali (*Ovis ammon*) (OUT, *n *=* *6) as an out‐group. After filtering, we obtained a dataset with 1119 SNPs, which was used for the TreeMix analysis. We added migration edges to the tree sequentially in order to choose the most frequently found variants with significant gene flow (*p* < 0.05). Standard errors (−*SE*) and *p*‐values were calculated using jackknife blocks of 10 SNPs (−*k* 10). We did not observe significant results when more than two migration events were allowed. To estimate how well our model fit the data, we inspected residuals from the trees. Residuals above zero indicate that populations are closer to each other than they are presented in the tree, and residuals below zero indicate that populations are less close to each other than they are presented in the tree. When no migration events were allowed, it was shown that MOM was the closest population to the out‐group (Figure 6a). However, residual fit indicated that TIK is more closely related to OUT than was presented in the best‐fit tree (Figure 6b). The model with two migration events (Figure 6c) revealed significant gene flow from the putative ancestral group, which existed before divergence of the studied populations and was related to the out‐group, to TIK (*p* = 0.015) and from the MOM/SKH common ancestral population to ORU (*p* = 0.025). This fact implies that MOM/SKH and TIK originated from two different ancestors. The residual plot corresponding to this tree (Figure 6d) indicated that MOM and SKH, as well as SKH and VER, were slightly genetically closer than they were presented in the tree, demonstrating that VER was also involved in the gene flow from MOM/SKH to ORU.

### 3.5 The f3 statistics

To clarify the history of the studied populations, we applied the f3 statistic as implemented in TreeMix 1.13 software. The f3 statistic (Reich et al., 2009) is a test for treeness in three population trees of the shape (X; A, B), which allows one to determine whether population X (target) is the result of admixture between A (source 1) and B (source 2). Negative *Z‐*scores from this statistic imply that the population X is admixed. We used jackknife blocks of 10 SNPs to detect the evidence of admixture in the studied populations of snow sheep. Significantly negative *Z*‐scores (*p* < 0.05) were observed for the (ORU; TIK, MOM) and (ORU; TIK, SKH) topologies, implying that ORU is derived from two ancestral populations (Table 1). Thus, the f3 statistic confirms that MOM and TIK have different origins and that the Orulgan Ridge is the territory where admixture between the two groups occurred.

**Table 1:** f3 statistics with significantly negative *Z*‐scores estimated in 112 blocks of size 10

Target	Source 1	Source 2	f3 statistic value	f3 statistic *SE*	*Z*‐score	*p*‐Value
ORU	TIK	MOM	−0.00187866	0.000907259	−2.07	0.038
ORU	TIK	SKH	−0.00131844	0.000643755	−2.05	0.041
ORU	TIK	VER	−0.00107231	0.000608251	−1.76	0.078

### 3.6 Genetic diversity

The observed (Ho) and expected (He) heterozygosity, inbreeding coefficient (*F*_IS_), and allelic richness (Ar) are shown in Table 2. The overall values of Ho and He (±*SE*) were 0.223 ± 0.005 and 0.243 ± 0.005, respectively. We found that the heterozygosity levels in populations of snow sheep increased southward; the minimum values were observed in the most northern population—TIK, and the maximum values were found in the most southern population—SKH. The distribution of heterozygosity at the individual level (sMLH) is presented in Supporting Information Table S2 and Figure S4. *F*_IS_ calculated for all the populations together (OVL) revealed a significant deficiency of heterozygotes in the snow sheep (*F*_IS_ 95% CI > 0), while for each group separately, the 95% CI included values of 0. The Ar values showed that the number of different heterozygous alleles in SKH was slightly lower than that in all the studied groups together. The lowest value of Ar was observed in TIK, and as in the case of heterozygosity, its level increased southward. The heterozygosity and allelic richness in the MOM population, which inhabits a different ridge in the east, were similar to those of ORU, with which they share the same latitude.

**Table 2:** Genetic diversity in the populations of snow sheep based on 1,121 SNP markers

Pop	*n*	Ho (±*SE*)	He (±*SE*)	*F*_IS_ [95% CI]	Ar (±*SE*)	Census size (*K*)	Climate^a^ January/July
TIK	22	0.201 ± 0.006	0.201 ± 0.006	0.006 [−0.009; 0.022]	1.570 ± 0.014	0.3–0.5	−31.0/7.4
ORU	22	0.223 ± 0.006	0.226 ± 0.006	0.014 [−0.000; 0.027]	1.667 ± 0.012	10–11	−47.3/15.9
VER	15	0.235 ± 0.006	0.237 ± 0.006	0.009 [−0.006; 0.024]	1.709 ± 0.012	10–11	−40.9/17.3
SKH	13	0.248 ± 0.006	0.247 ± 0.006	−0.002 [−0.017; 0.013]	1.733 ± 0.012	10.5–11.5	−46.0/14.7
MOM	8	0.227 ± 0.007	0.223 ± 0.006	−0.011 [−0.032; 0.011]	1.649 ± 0.014	8–9	−44.1/15.9
In total	80	0.223 ± 0.005	0.243 ± 0.005	0.074 [0.064; 0.083]	1.745 ± 0.009	38.8–43	−

a Average temperatures from the nearest meteorological stations are given: TIK—Tiksi, ORU—Verkhoyansk, VER—Batamai, SKH—Oymyakon, MOM—Ust‐Moma (http://climatebase.ru/stations/Russia/Yakutia).

## 4 DISCUSSION

Snow sheep (*Ovis nivicola* Eschscholtz, 1829) is the least genetically studied species within the Pachyceros subgenus, which also includes Dall and Stone sheep (*Ovis dalli*) and Rocky Mountain and desert bighorn sheep (*Ovis canadensis*) inhabiting North America. Due to difficult accessibility of the snow sheep habitat, sample collection is problematic, and such studies are limited. To date, Bunch et al. (2006) performed phylogenetic analysis of snow sheep collected in the Magadan region based on the mitochondrial cytochrome *b* of only four individuals. Rezaei et al. (2010) used three cytochrome *b* sequences of the samples taken from the aforementioned work to investigate the molecular phylogenetics and evolution of the genus *Ovis*. Danilkin (2005) sequenced the mtDNA control region, which had a length of 713 bp, of 40 samples from different habitats, such as the Kamchatka Peninsula, Koryak Mountains, Chukotka Autonomous District, Putorana Plateau, Verkhoyansk Range, and Kodar Ridge to clarify the phylogeny of snow sheep. Deniskova et al. (2016) demonstrated the successful application of microsatellites (STR) and the OvineSNP50 BeadChip designed for domestic sheep in evaluations of snow sheep.

In our study, we examined 80 samples of snow sheep collected in different ridges of Yakutia, namely Kharaulakh and Orulgan ridges, the central part of Verkhoyansk Range, Suntar‐Khayata Ridge, and Momsky Ridge, using the Illumina OvineSNP50 BeadChip developed for domestic sheep. The number of snow sheep inhabiting these territories is approximately 40 thousand individuals, which exceeds half of the census size of the species. According to the latest estimates of snow sheep census size in the Verkhoyansk Mountain chain, there were 300–500 individuals in Kharaulakh Ridge, approximately 10–11 thousand individuals in Orulgan Ridge, 10–11 thousand individuals in the central part of Verkhoyansk Range, and 10.5–11.5 thousand individuals in Suntar‐Khayata Ridge (Krivoshapkin, 2011). There is no precise information about the total number of snow sheep inhabiting Momsky Ridge, as this is a very remote and hard‐to‐reach area. Presumably, there are approximately 8‐9 thousand individuals (Revin, Sopin, & Zheleznov, 1988). One of the concerns of using SNP chips for cross‐species analysis could be the differences in karyotypes between the species for which the chip was developed and nonmodel organisms. In our case, *O. aries* and *O. nivicola* differ in chromosome number, with 54 and 52, respectively. However, the chromosome patterns in these species are similar, and the decrease in the number of chromosomes in *O. nivicola* arose from the centric fusion of the acrocentric chromosomes 9 and 19 (Bunch et al., 2006; Menscher, Bunch, & Maciulis, 1989).

The other concern could be the ascertainment bias that might affect the population assignment and assessment of genetic diversity parameters. This problem may occur in the analysis of joined datasets combining the breeds used for the development of SNP panels and those which were not taken into account during the construction of the DNA chips (Albrechtsen, Nielsen, & Nielsen, 2010; Nielsen, Hubisz, & Clark, 2004). We believe this should not preclude the use of the OvineSNP50 BeadChip in studies of snow sheep because neither of the *O*. *nivicola* groups was used for the creation of the used SNP panel. Additionally, introgression between *O. nivicola* and *O. aries,* which could also cause the ascertainment bias, could be excluded due to the absence of sheep husbandry in the areas of snow sheep habitat.

**Figure 7:**
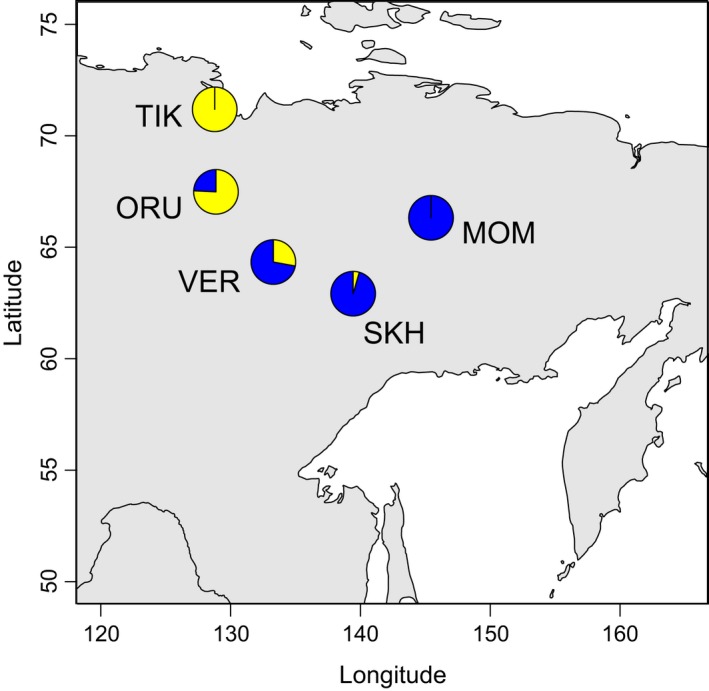
Spatial population admixture at *K *=* *2 in snow sheep inhabiting Verkhoyansk Mountains and Momsky Ridge

After quality control procedures, 1121 polymorphic SNPs distributed on all the autosomes were selected for subsequent analyses. Various approaches were used to evaluate the population structure of snow sheep. First, multidimensional scaling (MDS) showed that all the samples in our study were clustered according to their geographic locations (Figure 2). This was supported by the construction of the individual Neighbor‐Net tree (Supporting Information Figure S2). Then, model‐based individual ancestry estimation was performed to reveal the level of admixture between the populations. At *K *=* *2, which was determined as the most likely number of distinguishing groups in our subset by calculating cross‐validation error, the assignment of the studied populations to the two clusters was as follows: TIK—100% and 0%, ORU—75.7% and 24.3%, VER—29.7% and 70.3%, SKH—4.4% and 95.6%, and MOM—0% and 100% to the first and the second clusters, respectively (Figure 7). This allowed us to suggest that TIK and MOM have either different ancestral origins or long‐term isolation, and the three remaining populations are characterized by different levels of admixture between them. At *K *=* *5, when the number of clusters was assumed to be the number of populations in our study, we examined the way the populations were admixed with each other. TIK (99.4%) and MOM (100%) showed no traces of admixture with the other populations. In TIK, only an insignificant (95% CI) component of ORU (0.6%) was found. ORU (84.3%) was significantly (95% CI) admixed with TIK (10.5%) and VER (5.1%). A higher level of admixture with TIK was observed for the most northern ORU specimens. In VER (94.9%) and SKH (96.6%), only insignificant (95% CI) components of the other populations were found. Such results show a low level of gene flow between the populations of snow sheep. This may be explained by the fact that snow sheep are nonmigratory animals.

This was supported by calculations of pairwise *F*_ST_ values, which showed significant (*p* < 0.001) genetic differentiation among all the populations of snow sheep from low (*F*_ST_ < 0.05) between adjacent populations to moderate (*F*_ST_ < 0.15) and even high between the most distant groups TIK and MOM (*F*_ST_ = 0.205). In addition, the evaluation of isolation by distance (IBD), performed with a Mantel test (Figure 5), demonstrated an extremely high correlation (*r* ^2 ^= 0.979, *p* = 0.009) between the pairwise *F*_ST_ values and geographic distances (km).

The history of divergence and admixtures in the studied populations of snow sheep was elucidated using the f3 statistic and TreeMix analysis in which argali (*Ovis ammon*) was added to our dataset as an out‐group. It has been discovered that TIK has a different origin from MOM, SKH, and VER. ORU was characterized as an admixed population. Most likely, TIK was isolated from the other groups of snow sheep during the periods of Late Pleistocene glaciation of Verkhoyansk Range. Glaciers in this region attained their maximum extent approximately 140 kya, and further series of glaciations continued up to 50 kya. Ice masses were generally mountain‐centered and did not extend more than 270 km beyond the ridges (Barr & Clark, 2012; Hughes, Gibbard, & Ehlers, 2013; Stauch, Lehmkuhl, & Frechen, 2007). Apparently, the low‐altitude Kharaulakh Mountains played the role of a glacial refugium for a population of snow sheep. When the last melting of the glaciers took place in Verkhoyansk Range approximately 50 kya, its area was recolonized by populations that were inhabiting glacier‐free territories in the south and southeast and by the population from the Kharaulakh Mountains. After several thousands of years of separate habitat, the groups of snow sheep met in Orulgan Ridge and gave rise to an admixed population—ORU. These results demonstrate the major role of glaciation events in the genetic diversity and distribution of subarctic species, such as snow sheep, and agree with the investigations on the survival of a group of thin‐horn sheep (*Ovis dalli*) in a minor refugium (Sim, Hall, Jex, Hegel, & Coltman, 2016).

As long as genetic diversity can affect the productivity, growth, and stability of the focal population, the estimation of its parameters, such as observed and expected heterozygosity, allelic richness, and the inbreeding coefficient, is essential for conservation programs (Hughes, Inouye, Johnson, Underwood, & Vellend, 2008). In our study, SNP analysis revealed that the measures of allelic richness and observed and expected heterozygosity in the populations of snow sheep increased southward. This can be explained by the fact that these parameters are influenced by rates of gene flow and genetic drift. TIK, which is characterized by the lowest heterozygosity values, is found at the edge of the species distribution. For this population, only gene transfer with ORU is possible, and the decrease in diversity probably occurred due to the loss of rare alleles. The highest level of heterozygosity was detected in SKH, which is situated at the intersection of different ridges including Central Verkhoyansk, Chersky, and Sette‐Dabban, and animals from various populations may admix in this territory. Such admixture allows the maintenance of genetic diversity parameters at a higher level.

The inbreeding coefficient (*F*_IS_) in each group was not significantly different from zero (95% CI), indicating that the populations were neither inbred nor outbred. However, the calculation of *F*_IS_ for the entire subset of individuals altogether showed a significantly moderate level of inbreeding in snow sheep. This fact reveals that for snow sheep and other species with a clear population structure and different levels of heterozygosity between the groups, the calculation of *F*_IS_ may produce misleading results if samples are taken from remote locations.

## 5 CONCLUSIONS

Our research revealed that the studied populations of snow sheep are genetically structured according to a geographic pattern. Genetic diversity parameters (observed and expected heterozygosity and allelic richness) were increasing southward, with minimum values in the most northern population—TIK and maximum values in the most southern population—SKH. It was found that snow sheep inhabiting territories in Verkhoyansk Range and Momsky Ridge originated from two different ancestral populations even though they are conventionally considered a single subspecies, known as Yakut snow sheep (*Ovis nivicola lydekkeri*). The studied population from Kharaulakh Ridge (TIK) represent descendants of the group that was isolated from other groups of snow sheep for a long time, most likely during the time of glaciation of northeastern Siberia. A mixture between this population and migrants from the other studied populations occurred in the Orulgan Ridge. As long as the numbers of Kharaulakh snow sheep are rather small and they inhabit a limited territory that is located in a low‐hill terrain, making them easy prey for poachers, we suggest that this group should be treated with special care. As one of the solutions, we propose to classify this group as a separate subspecies.

## CONFLICT OF INTEREST

None declared.

## AUTHOR CONTRIBUTIONS

AD, NZ, and GB conceived the study design and specified the research goals; AD, TD, and GM wrote the manuscript; AD and HR provided data processing and analytical support; TD and HR performed molecular genetic studies; IO provided the samples; AD, TD, GM, IO, HR, KW, JS, GB, and NZ developed the manuscript concept and contributed to the discussion. All authors read and approved the final manuscript.

## DATA ACCESSIBILITY

Data available from the Dryad Digital Repository: https://doi.org/10.5061/dryad.ps8kr80

## References

[ece34350-bib-0001] Albrechtsen, A. , Nielsen, F. C. , & Nielsen, R. (2010). Ascertainment biases in SNP chips affect measures of population divergence. Molecular Biology and Evolution, 27(11), 2534–2547. 10.1093/molbev/msq148 20558595 PMC3107607

[ece34350-bib-0002] Alexander, D. H. , Novembre, J. , & Lange, K. (2009). Fast model‐based estimation of ancestry in unrelated individuals. Genome Research, 19, 1655–1664. 10.1101/gr.094052.109 19648217 PMC2752134

[ece34350-bib-0003] Barr, I. D. , & Clark, C. D. (2012). Late Quaternary glaciations in Far NE Russia; Combining moraines, topography and chronology to assess regional and global glaciation synchrony. Quaternary Science Reviews, 53, 72–87. 10.1016/j.quascirev.2012.08.004

[ece34350-bib-0004] Becker, R. A. , Wilks, A. R. , Brownrigg, R. , Minka, T. P. , & Deckmyn, A. (2017). maps: Draw geographical maps. R package version 3.2.0. Retrieved from https://CRAN.R-project.org/package=maps

[ece34350-bib-0005] Bunch, T. D. , Wu, C. , Zhang, Y. P. , & Wang, S. (2006). Phylogenetic analysis of snow sheep (*Ovis nivicola*) and closely related taxa. Journal of Heredity, 97, 21–30. 10.1093/jhered/esi127 16267166

[ece34350-bib-0006] Campbell, A. , Kapos, V. , Scharlemann, J. P. W. , Bubb, P. , Chenery, A. , Coad, L. , … Rashid, M. (2009). Review of the literature on the links between biodiversity and climate change: Impacts, adaptation and mitigation. Secretariat of the Convention on Biological Diversity, Montreal. Technical Series No. 42, 124 pages

[ece34350-bib-0007] Coates, B. S. , Sumerford, D. V. , Miller, N. J. , Kim, K. S. , Sappington, T. W. , Siegfried, B. D. , & Lewis, L. C. (2009). Comparative performance of single nucleotide polymorphism and microsatellite markers for population genetic analysis. Journal of Heredity, 100, 556–564. 10.1093/jhered/esp028 19525239

[ece34350-bib-0008] Coltman, D. W. , Pilkington, J. G. , Smith, J. A. , & Pemberton, J. M. (1999). Parasite‐mediated selection against inbred Soay sheep in a free‐living, island population. Evolution, 53, 1259–1267. 10.1111/j.1558-5646.1999.tb04538.x 28565537

[ece34350-bib-0009] Danilkin, A. A. (2005). Mammals of Russia and Adjacent Regions: Hollow‐horned ruminants (Bovidae). Moscow, Russia: KMK (in Russian)

[ece34350-bib-0010] Deniskova, T. E. , Sermyagin, A. A. , Bagirov, V. A. , Okhlopkov, I. M. , Gladyr, E. A. , Ivanov, R. V. , … Zinovieva, N. A. (2016). Comparative analysis of the effectiveness of STR and SNP markers for intraspecific and interspecific differentiation of the genus Ovis. Russian Journal of Genetics, 52, 79–84. 10.1134/S1022795416010026 27183797

[ece34350-bib-0011] Francis, R. M. (2017). pophelper: An R package and web app to analyse and visualize population structure. Molecular Ecology Resources, 17, 27–32. 10.1111/1755-0998.12509 26850166

[ece34350-bib-0012] Frichot, E. , & François, O. (2015). LEA: An R package for landscape and ecological association studies. Methods in Ecology and Evolution, 6, 925–929. 10.1111/2041-210X.12382

[ece34350-bib-0013] Gerritsen, H. (2014). mapplots: Data visualisation on maps. R package version 1.5. Retrieved from https://CRAN.R-project.org/package=mapplots

[ece34350-bib-0014] Haynes, G. D. , & Latch, E. K. (2012). Identification of novel single nucleotide polymorphisms (SNPs) in deer (*Odocoileus* spp.) using the BovineSNP50 BeadChip. PLoS ONE, 7, e36536 10.1371/journal.pone.0036536 22590559 PMC3348150

[ece34350-bib-0015] Hijmans, R. J. (2017). geosphere: Spherical Trigonometry. R package version 1.5‐7. Retrieved from https://CRAN.R-project.org/package=geosphere

[ece34350-bib-0016] Hughes, P. D. , Gibbard, P. L. , & Ehlers, J. (2013). Timing of glaciation during the last glacial cycle: Evaluating the concept of a global ‘Last Glacial Maximum’ (LGM). Earth‐Science Reviews, 125, 171–198. 10.1016/j.earscirev.2013.07.003

[ece34350-bib-0017] Hughes, A. R. , Inouye, B. D. , Johnson, M. T. J. , Underwood, N. , & Vellend, M. (2008). Ecological consequences of genetic diversity. Ecology Letters, 11, 609–623. 10.1111/j.1461-0248.2008.01179.x 18400018

[ece34350-bib-0018] Huson, D. H. , & Bryant, D. (2006). Application of phylogenetic networks in evolutionary studies. Molecular Biology and Evolution, 23, 254–267. 10.1093/molbev/msj030 16221896

[ece34350-bib-0019] Kalinowski, S. T. , Wagner, A. P. , & Taper, M. L. (2006). ML‐Relate: A computer program for maximum likelihood estimation of relatedness and relationship. Molecular Ecology Notes, 6, 576–579. 10.1111/j.1471-8286.2006.01256.x

[ece34350-bib-0020] Keenan, K. , McGinnity, P. , Cross, T. F. , Crozier, W. W. , & Prodöhl, P. A. (2013). diveRsity: An R package for the estimation and exploration of population genetics parameters and their associated errors. Methods in Ecology and Evolution, 4, 782–788. 10.1111/2041-210X.12067

[ece34350-bib-0021] Kharzinova, V. R. , Sermyagin, A. A. , Gladyr, E. A. , Okhlopkov, I. M. , Brem, G. , & Zinovieva, N. A. (2015). A study of applicability of SNP chips developed for Bovine and Ovine species to whole‐genome analysis of Reindeer Rangifer tarandus. Journal of Heredity, 106, 758–761. 10.1093/jhered/esv081 26447215

[ece34350-bib-0022] Kijas, J.W. , Lenstra, J.A. , Hayes, B. , Boitard, S. , Porto Neto, L.R. , San Cristobal, M. , & International Sheep Genomics Consortium Members (2012). Genome‐wide analysis of the world's sheep breeds reveals high levels of historic mixture and strong recent selection. PLoS Biology, 10(2), e1001258 10.1371/journal.pbio.1001258 22346734 PMC3274507

[ece34350-bib-0023] Kijas, J. W. , Townley, D. , Dalrymple, B. P. , Heaton, M. P. , Maddox, J. F. , McGrath, A. , & the International Sheep Genomics Consortium. (2009). A genome wide survey of SNP variation reveals the genetic structure of sheep breeds. PLoS ONE, 4, e4668 10.1371/journal.pone.0004668 10.1371/journal.pone.0004668 19270757 PMC2652362

[ece34350-bib-0024] Kohn, M. H. , Murphy, W. J. , Ostrander, E. A. , & Wayne, R. K. (2006). Genomics and conservation genetics. Trends in Ecology and Evolution, 21, 629–637. 10.1016/j.tree.2006.08.001 16908089

[ece34350-bib-0025] Krivoshapkin, A. A. (2011). The current state of the snow sheep *(Ovis nivicola Esch.)* census size in the Verkhoyansk mountain system. Vestnik of NEFU, 8, 17–21. (in Russian).

[ece34350-bib-0026] Mantel, N. (1967). The detection of disease clustering and a generalized regression approach. Cancer Research, 27, 209–220. 6018555

[ece34350-bib-0027] Menscher, S. H. , Bunch, T. D. , & Maciulis, A. (1989). High‐resolution G‐banded karyotype and idiogram of the goat: A sheep‐goat G‐band comparison. Journal of Heredity, 80, 150–155. 10.1093/oxfordjournals.jhered.a110816 2926117

[ece34350-bib-0028] Mergeay, J. , & Santamaria, L. (2012). Evolution and Biodiversity: The evolutionary basis of biodiversity and its potential for adaptation to global change. Evolutionary Applications, 5(2), 103–106. 10.1111/j.1752-4571.2011.00232.x 25568033 PMC3353341

[ece34350-bib-0029] Miller, J. M. , Kijas, J. W. , Heaton, M. P. , McEwan, J. C. , & Coltman, D. W. (2012). Consistent divergence times and allele sharing measured from cross‐species application of SNP chips developed for three domestic species. Molecular Ecology Resources, 12, 1145–1150. 10.1111/1755-0998.12017 22994965

[ece34350-bib-0030] Miller, J. M. , Poissant, J. , Kijas, J. W. , Coltman, D. W. , & and the International Sheep Genomics Consortium (2011). A genome‐wide set of SNPs detects population substructure and long range linkage disequilibrium in wild sheep. Molecular Ecology Resources, 11, 314–322. 10.1111/j.1755-0998.2010.02918.x 21429138

[ece34350-bib-0031] Morin, P. A. , Luikart, G. , Wayne, R. K. , & the SNP workshop group. (2004). SNPs in ecology, evolution and conservation. Trends in Ecology and Evolution, 19, 208–216. 10.1016/j.tree.2004.01.009 10.1016/j.tree.2004.01.009

[ece34350-bib-0032] Nei, M. (1978). Estimation of average heterozygosity and genetic distance from a small number of individuals. Genetics, 83, 583–590. 10.1093/genetics/89.3.583 PMC1213855 17248844

[ece34350-bib-0033] Nielsen, R. , Hubisz, M. J. , & Clark, A. G. (2004). Reconstituting the frequency spectrum of ascertained single‐nucleotide polymorphism data. Genetics, 168, 2373–2382. 10.1534/genetics.104.031039 15371362 PMC1448751

[ece34350-bib-0034] Ogden, R. (2011). Unlocking the potential of genomic technologies for wildlife forensics. Molecular Ecology Resources, 11, 109–116. 10.1111/j.1755-0998.2010.02954.x 21429167

[ece34350-bib-0035] Peakall, R. , & Smouse, P. E. (2012). GenAlEx 6.5: Genetic analysis in Excel. Population genetic software for teaching and research — an update. Bioinformatics, 28, 2537–2539. 10.1093/bioinformatics/bts460 22820204 PMC3463245

[ece34350-bib-0036] Pembleton, L. W. , Cogan, N. O. , & Forster, J. W. (2013). StAMPP: An R package for calculation of genetic differentiation and structure of mixed‐ploidy level populations. Molecular Ecology Resources, 13, 946–952. 10.1111/1755-0998.12129 23738873

[ece34350-bib-0037] Pickrell, J. K. , & Pritchard, J. K. (2012). Inference of population splits and mixtures from genome‐wide allele frequency data. PLoS Genetics, 8, e1002967 10.1371/journal.pgen.1002967 23166502 PMC3499260

[ece34350-bib-0038] Purcell, S. , Neale, B. , Todd‐Brown, K. , Thomas, L. , Ferreira, M. A. , Bender, D. , … Sham, P. C. (2007). PLINK: A tool set for whole‐genome association and population‐based linkage analyses. American Journal of Human Genetics, 81, 559–575. 10.1086/519795 17701901 PMC1950838

[ece34350-bib-0039] Reich, D. , Thangaraj, K. , Patterson, N. , Price, A. L. , & Singh, L. (2009). Reconstructing Indian population history. Nature, 461, 489–494. 10.1038/nature08365 19779445 PMC2842210

[ece34350-bib-0040] Revin, Y. V. , Sopin, L. V. , & Zheleznov, N. K. (1988). Snow sheep. Novosibirsk, Russia: Nauka. (in Russian).

[ece34350-bib-0041] Rezaei, H. R. , Naderi, S. , Chintauan‐Marquier, I. C. , Taberlet, P. , Virk, A. T. , Naghash, H. R. , … Pompanon, F. (2010). Evolution and taxonomy of the wild species of the genus Ovis (Mammalia, Artiodactyla, Bovidae). Molecular Phylogenetics and Evolution, 54, 315–326. 10.1016/j.ympev.2009.10.037 19897045

[ece34350-bib-0042] Seeb, J. E. , Carvalho, G. , Hauser, L. , Naish, K. , Roberts, S. , & Seeb, L. W. (2011). Single‐nucleotide polymorphism (SNP) discovery and applications of SNP genotyping in nonmodel organisms. Molecular Ecology Resources, 11, 1–8. 10.1111/j.1755-0998.2010.02979.x 21429158

[ece34350-bib-0043] Sim, Z. , Hall, J. C. , Jex, B. , Hegel, T. M. , & Coltman, D. W. (2016). Genome‐wide set of SNPs reveals evidence for two glacial refugia and admixture from postglacial recolonization in an alpine ungulate. Molecular Ecology, 25, 3696–3705. 10.1111/mec.13701 27272944

[ece34350-bib-0044] Stauch, G. , Lehmkuhl, F. , & Frechen, M. (2007). Luminescence chronology from the Verkhoyansk Mountains (North‐Eastern Siberia). Quaternary Geochronology, 2, 255–259. 10.1016/j.quageo.2006.05.013

[ece34350-bib-0045] Stoffel, M. A. , Esser, M. , Kardos, M. , Humble, E. , Nichols, H. , David, P. , & Hoffman, J. I. (2016). inbreedR: An R package for the analysis of inbreeding based on genetic markers. Methods in Ecology and Evolution, 7, 1331–1339. 10.1111/2041-210X.12588

[ece34350-bib-0046] Tokarska, M. , Marshall, T. , Kowalczyk, R. , Wójcik, J. M. , Pertoldi, C. , Kristensen, T. N. , … Bendixen, C. (2009). Effectiveness of microsatellite and SNP markers for parentage and identity analysis in species with low genetic diversity: The case of European bison. Heredity, 103, 326–332. 10.1038/hdy.2009.73 19623210

[ece34350-bib-0047] Vignal, A. , Milan, D. , SanCristobal, M. , & Eggen, A. (2002). A review on SNP and other types of molecular markers and their use in animal genetics. Genetics Selection Evolution, 34, 275–305. 10.1186/1297-9686-34-3-275 PMC2705447 12081799

[ece34350-bib-0048] Weir, B. S. , & Cockerham, C. C. (1984). Estimating F‐Statistics for the analysis of population structure. Evolution, 38, 1358–1370. 28563791 10.1111/j.1558-5646.1984.tb05657.x

[ece34350-bib-0049] Wickham, H. (2009). ggplot2: Elegant graphics for data analysis. New York, NY: Springer‐Verlag 10.1007/978-0-387-98141-3

[ece34350-bib-0050] Zheleznov‐Chukotsky, N. K. (1994). Ecology of snow sheep of Northern Asia. Moscow, Russia: Nauka. (in Russian).

[ece34350-bib-0051] Zheleznov‐Chukotsky, N. K. (2007). Project OVIS‐ZH‐CH “Conservation and increasing of population of snow sheep (Ovis nivicola Eschscholtz, 1829) in Russia” in 2007–2017. The Herald of Game Management, 4, 285–315. (in Russian).

