# Supplementary figures and images for: Genome‐wide SNP analysis unveils genetic structure and phylogeographic history of snow sheep (Ovis nivicola) populations inhabiting the Verkhoyansk Mountains and Momsky Ridge (northeastern Siberia)
Source: Ecol Evol. 2018 Jul 16;8(16):8000–10. doi: 10.1002/ece3.4350 (PMC6144981; doi:10.1002/ece3.4350)

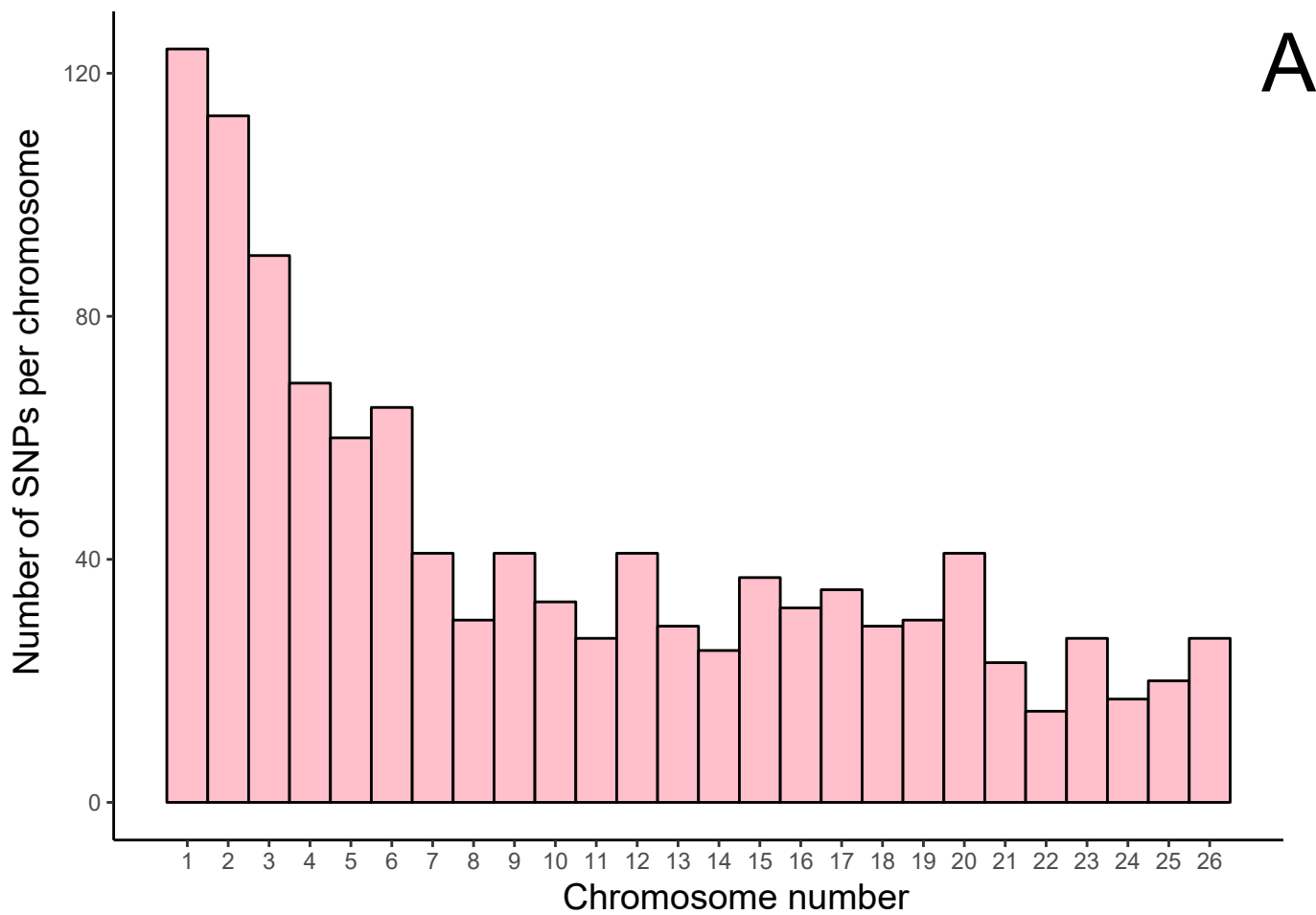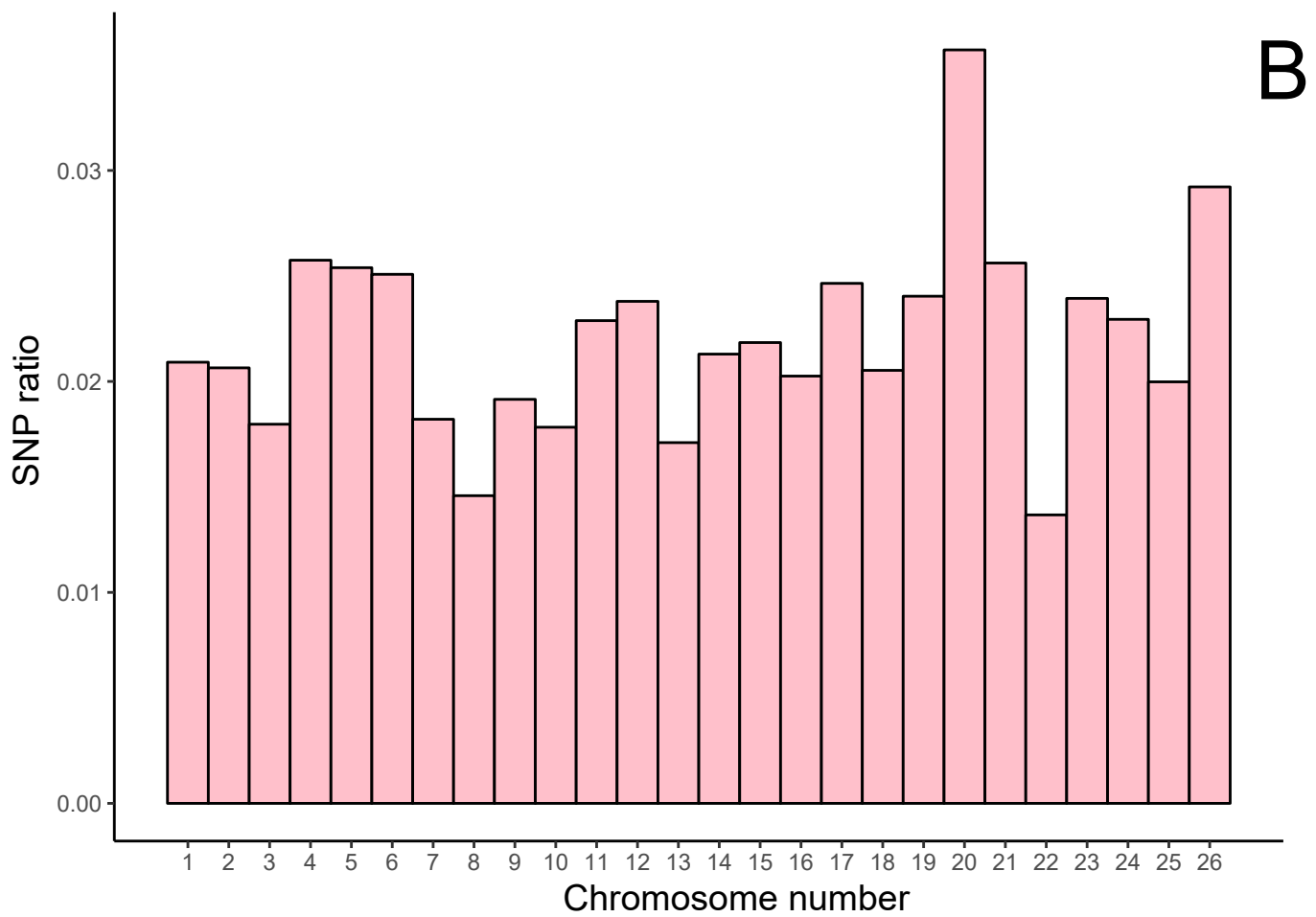

Supplement: Supplementary file 1 [file ECE3-8-8000-s001.pdf]

0.01

VER

ORU

MOM

SKH

TIK

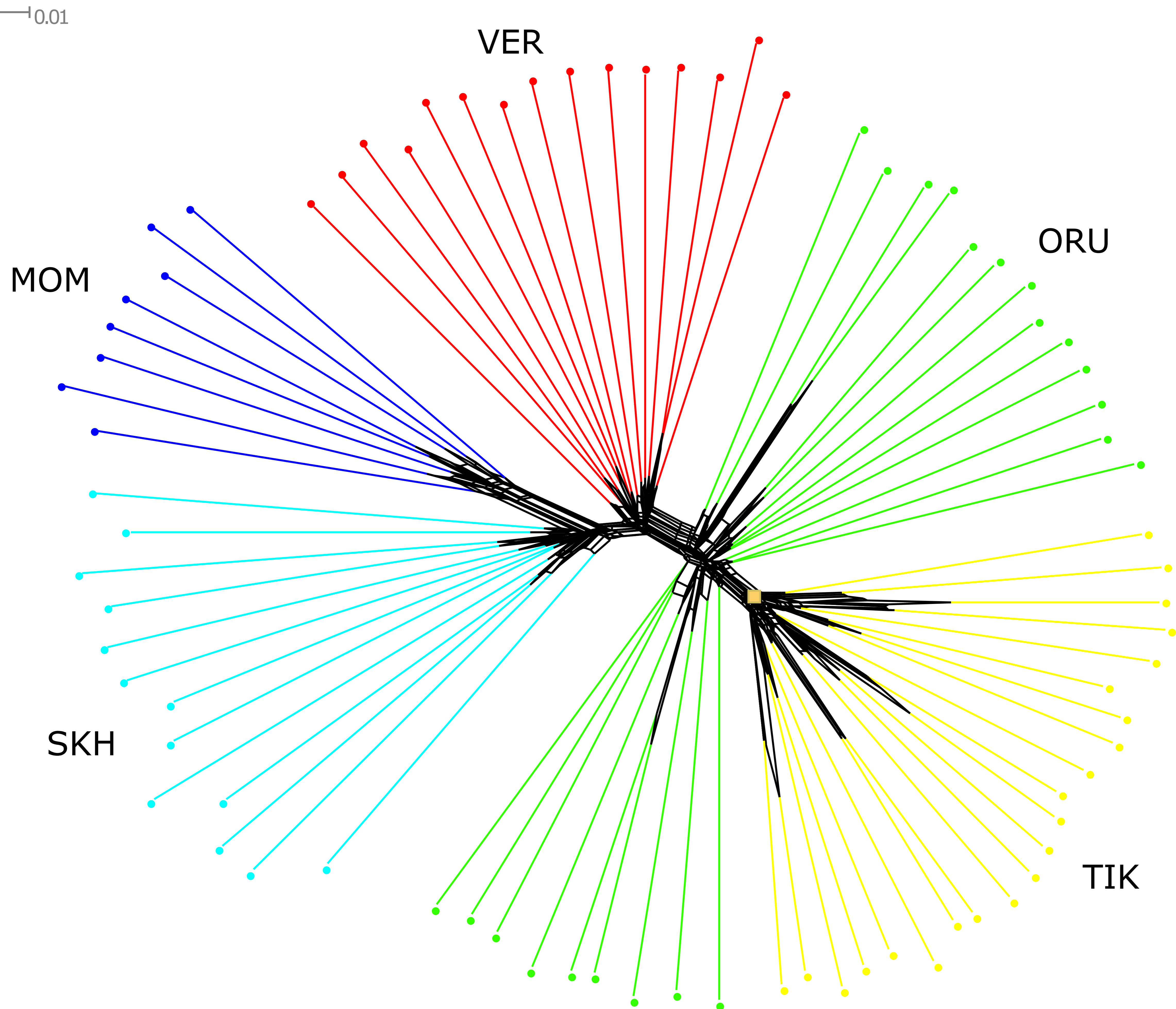

Supplement: Supplementary file 2 [file ECE3-8-8000-s002.pdf]

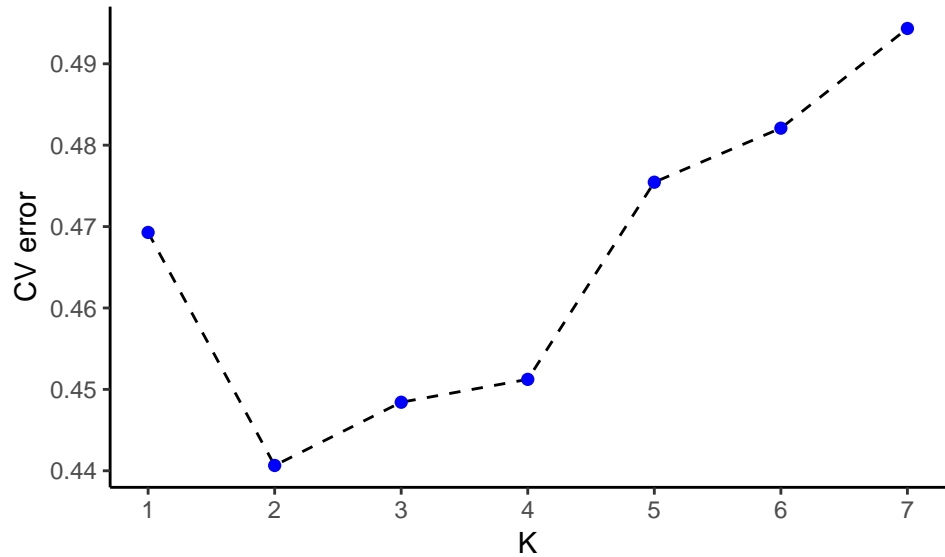

Supplement: Supplementary file 3 [file ECE3-8-8000-s003.pdf]

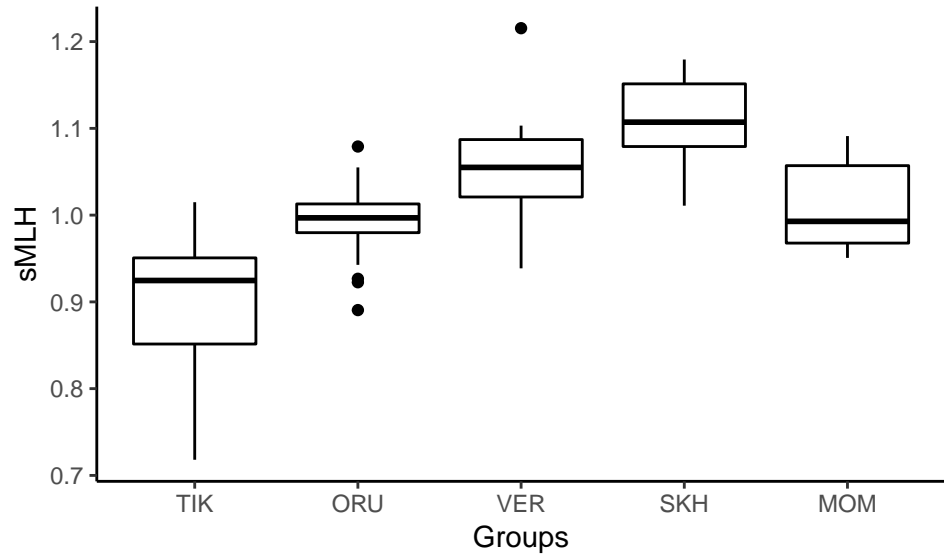

Supplement: Supplementary file 4 [file ECE3-8-8000-s004.pdf]
