# Supplementary material for: Genome‐wide SNP analysis unveils genetic structure and phylogeographic history of snow sheep (Ovis nivicola) populations inhabiting the Verkhoyansk Mountains and Momsky Ridge (northeastern Siberia)
Source: Ecol Evol. 2018 Jul 16;8(16):8000–10. doi: 10.1002/ece3.4350 (PMC6144981; doi:10.1002/ece3.4350)
Supplement: Supplementary file 5 [file ECE3-8-8000-s005.docx]

**Table S1. The matrix with pairwise *F*_ST_ values (below diagonal) and geographical distances (km) between the studied groups of snow sheep (above diagonal) used in calculations of the Mantel test.**

| Pop | TIK | ORU | VER | SKH | MOM |
| --- | --- | --- | --- | --- | --- |
| TIK | 0 | 418.5 | 809.7 | 1147.3 | 1610.9 |
| ORU | 0.052 | 0 | 391.2 | 728.8 | 1192.4 |
| VER | 0.112 | 0.053 | 0 | 337.6 | 801.2 |
| SKH | 0.144 | 0.086 | 0.044 | 0 | 463.6 |
| MOM | 0.205 | 0.142 | 0.111 | 0.076 | 0 |

**Table S2. Standardized multilocus heterozygosity (sMLH) in the populations of snow sheep.**

| Pop | n | sMLH | Range (min-max) |
| --- | --- | --- | --- |
| TIK | 22 | 0.900±0.017 | 0.718 – 1.015 |
| ORU | 22 | 0.994±0.010 | 0.891 – 1.079 |
| VER | 15 | 1.052±0.018 | 0.939 – 1.215 |
| SKH | 13 | 1.112±0.014 | 1.010 – 1.180 |
| MOM | 8 | 1.011±0.0.20 | 0.951 – 1.091 |
